# Gene, virulence and related regulatory mechanisms in *Cryptococcus gattii*: Gene, virulence and regulatory mechanisms in *C*. *gattii*

**DOI:** 10.3724/abbs.2022029

**Published:** 2022-03-25

**Authors:** Yemei Huang, Xuelei Zang, Chen Yang, Hengyu Deng, Xidong Ma, Mei Xie, Meng Zhou, Jialin Song, Xinying Xue

**Affiliations:** 1 Department of Respiratory and Critical Care Beijing Shijitan Hospital Capital Medical University; Peking University Ninth School of Clinical Medicine Beijing 100089 China; 2 Department of Laboratory Medicine the First Medical Centre Chinese PLA General Hospital Beijing 100853 China; 3 School of Clinical Medicine Weifang Medical University Weifang 261053 China; 4 Binzhou Medical University Binzhou 246003 China

**Keywords:** *Cryptococcus gattii*, genotype, virulence, regulation mechanism

## Abstract

*Cryptococcus gattii* is a kind of basidiomycetous yeast, which grows in human and animal hosts. *C*. *gattii* has four distinct genomes, VGI/AFLP4, VGII/AFLP6, VGIII/AFLP5, and VGIV/AFLP7. The virulence of *C*. *gattii* is closely associated with genotype and related stress-signaling pathways, but the pathogenic mechanism of *C*. *gattii* has not been fully identified. With the development of genomics and transcriptomics, the relationship among genes, regulatory mechanisms, virulence, and treatment is gradually being recognized. In this review, to better understand how *C*. *gattii* causes disease and to characterize hypervirulent *C*. *gattii* strains, we summarize the current understanding of *C*. *gattii* genotypes, phenotypes, virulence, and the regulatory mechanisms.

## Introduction

*Cryptococcus gattii* is a kind of pathogenic yeast that causes pulmonary infections and meningoencephalitis mainly in immunocompetent hosts, especially in tropical and sub-tropical regions [1], and is significantly more lethal than *C*. *Neoformans* [2]. Therefore, the studies of its virulence, related genes and pathways are essential for understanding and exploring therapeutic approaches. In the last two decades, the flourishing of molecular technologies has greatly facilitated the study of genomics, virulence phenotypes, and stress signaling pathways, allowing us to analyze the pathogenesis, potential biomarkers, safe and effective therapeutics, and vaccines of *C*. *gattii* [ 3, 4].

This review summarizes the logical relationship among genotype, transcription, virulence phenotype and regulatory mechanisms about *C*. *gattii* in a historical context.

## Genetic Characteristics of *C*. *gattii*

### Taxonomy and distribution

*C*. *gattii* is classified into four main genetic molecular species: *C*. *gattii* (VGI), *C*. *deuterogattii* (VGII with subtypes VGIIa, VGIIb, and VGIIc), *C*. *bacillisporus* (VGIII), and *C*. *tetragattii* (VGIV), and some of the rarer branches are associated with these lineage-related rarer branches (VGIV/VGIIIc, VGV), each containing both B and C serotypes [ 5– 7]. PCR fingerprint analysis of four loci, *ACT1*, *IDE1*, *PLB1* and *URA5*, confirmed that VGII is the oldest lineage of *C*. *gattii* [8]. The analysis of clinical, animal and environmental isolate strains also showed that the most common molecular type is VGII (47%), followed by VGI (34%), VGIII (11%) and VGIV (8%); most of the environmental isolates are molecular type VGII, followed by VGI, VGIII and VGIV [ 4, 9]. Furthermore, the distribution of VGI–VGIV varies by regions: VGI isolates are common in Asia, Australia, and Europe, whereas isolates of VGII have been well sampled by sequencing to understand the recent outbreak in the Pacific Northwest. In addition, VGIII is widely distributed in South America, such as Mexico, Colombia, and sporadic cases are recorded in Africa; VGIV is most prevalent in India and Africa; and taken together, *C*. *gattii* occurs primarily in more temperate climates [ 5, 10– 12]. Early genetic sequencing analyses focused mainly on single or small gene loci; in 2009, the International Society for Human and Animal Mycology (ISHAM) Cryptococcus Working Group agreed upon Multilocus sequence typing (MLST) as the standardized genotyping approach. Since then, MLST has been used in *C*. *gattii* studies and has yielded substantial and reproducible results between laboratories [5]. Previous amplified fragment length polymorphism (AFLP) and MLST studies have demonstrated that the Vancouver Island outbreak (VIO) is mainly caused by a single, hypervirulent genotype of *C*. *gattii* (AFLP6A/molecular type VGIIa) [13]. A previous study based on MLST showed that the VGII lineage itself originated ancestrally from South America [14]; however, the origin of the outbreak strain remains unknown. Both Australia [15] and South America [ 14, 15] have been suggested as possible geographical origins [16].

Although all four lineages of *C*. *gattii* can cause disease, VGI and VGII cause most infections in immunocompetent hosts, while infection in the VGIII and VGIV groups is rare and occurs mainly in immunocompromised hosts [1]. Previous studies have shown that *C*. *gattii* strains isolated from HIV/AIDS patients in Africa and America are almost entirely VGIII and VGIV, and the main reason may be related to the difference in virulence among different molecular subtypes [ 1, 17– 19]. Another study has shown that VGII, VGIII, and VGIV have a lower median survival rates, larger capsules and higher capsule percentage than VGI, suggesting differences in virulence among strains with different phenotypes [20]. In terms of virulence phenotypes, the VGI strain has the largest capsules but smaller cells among the four phenotypes, while VGII strain has the largest cells but smaller capsules among all phenotypes. At the same time, the overall heat resistance of VGII strain is significantly higher than that in the other three types, especially at 37°C, and the growth of VGIII and VGIV strains is significantly reduced [21]. The results of virulence tests on rats also suggested that VGIII and VGIV have different virulences, with VGIV being able to produce a large number of fungi in the lungs with a high mortality rate, while VGIII is less virulent and cannot reach the brain to cause disease [22]. However, another study showed the highest virulence of VGIII in a *Drosophila* model, suggesting differences in virulence between different phenotypes in different hosts [23]. Although all four lineages are capable of causing disease, a number of differences have been identified among sub-lineages, such as the enhanced ability of VGIIa outbreak isolates to parasitize host phagocytes; these processes are initiated upon macrophage phagocytosis, followed by a stress response that triggers cryptococcal mitochondrial tubularization and rapid proliferation of the outbreak strains [24]. In a study of two VGII subtypes (a and b) isolated from the Vancouver outbreak, microarray analysis showed that genes encoding putative virulence factors (such as *LAC1*, *LAC2*, *CAS3*, and *MPK1*) and genes encoding cell wall assembly proteins are increased in strain R265, while genes involved in the regulation of mitosis and ergosterol biosynthesis are decreased [25]. In another study, VGIIb, α-α and a-α mating events occurred in VGIIa and VGIIc, and highly tubular mitochondria were formed after parasitism in cells, with higher virulences were compared [26], suggesting that differences in virulence phenotype may be related to different gene expressions.

### Gene evolution and virulence

Genetic studies have focused more on the links between pathogenic virulence and gene evolution, in preparation for future outbreaks.

Except for molecular species, there are some studies on gene evolution of expression and phenotype, including gene mutants and chromosome structural changes (chromosome rearrangements and disomic copy), which account for higher virulence. In gene mutants, the changes in virulence phenotypes in two isolates of VGIIc, EJB52 and EJB18 (intracellular active rates and mitochondrial tubularization rates and phagocytosis patterns) are significantly associated with two nuclear gene variants, the gene insertion (non-frameshift/modulo 3) and non-synonymous change (T→C, I→V) [24]. Blake *et al*. [16] used whole genome sequencing to determine the potential causes of increased virulence in VGII outbreak isolates. The results showed that VGIIa underwent genetic transformation and mitotic microevolution driven by mutant phenotype (a total of twelve missense mutations were identified, but only one shift mutation), leading to a significant increase in virulence [16]. Among the chromosome structural changes, a total of 15 large (>100 kb) chromosomal rearrangements were identified in the four lineages (VGI–VGIV), and on average, only 2.6% of the 16 genomes were rearranged in relation to other genomes. The results of these gene amplifications/contractions and positive selection may affect the ability of genetic exchange between lineages and diversify the pathogenic mechanisms of the complex [1]. Similarly, studies of the initial *C*. *gattii* strains from the Vancouver Island outbreak showed that following natural events involving rearrangements between two ancestral strains, the resulting progeny are more virulent than the parental strain [15]. Moreover, researchers have identified additional (dimorphic) copies of scaffold 13 (SC13) in VGII veterinary isolate B8828 and dimorphic copies of SCII in VGIII clinical isolate CA1280 (syntenic to the first half of WM276 chromosome cgba), which may have higher virulence [1]. In all these studies, the microevolution of *C*. *gattii* genes results in increased virulence, but there is still much room to explore the real contributing gene transformation and its related mechanisms.

### Mitochondrial gene and virulence

What is more, mitochondrial regions are very useful genetic markers for pathopoiesis, as mitochondria evolve independently of the nuclear genome, thus providing additional independent datasets [27]. It is extensively explored in *C*. *Neoformans* that mitochondria are usually uniparentally inherited from the MATa parent [27]. While in *C*. *gattii*, fungal gene expression in host macrophages was compared between hypervirulent (VIO) and hypovirulent (non-VIO) strains using microarray approaches, and mitochondrial tubularization of VIO strain showed enhanced intracellular proliferation of the strain within host macrophage cells [28]. The intracellular proliferative capacity of macrophages is positively correlated with mitochondrial tubulation (linear regression *P*<0.0001, *n*=24), which is not observed in *C*. *Neoformans* [29]. Taken together, mitochondrial tubularization is correlated with intracellular proliferative potential and is a specific feature of *C*. *gattii* outbreak strain. In *C*. *Neoformans* and *C*. *gattii*, the group I introns are structural sequences capable of catalyzing their splicing from precursor RNA in the mitochondrial large subunit rRNA gene (LSU rRNA), and it was reported that intron presence might be associated with higher virulence and higher MIC ( *P*<0.001), suggesting the relationship between mitochondrial genotypes and virulence [30]. Therefore, pathogenic mitochondrial regulation may be a common and important factor, and more experiments are needed to testify its role.

## Virulence Factors

**Figure 1:**
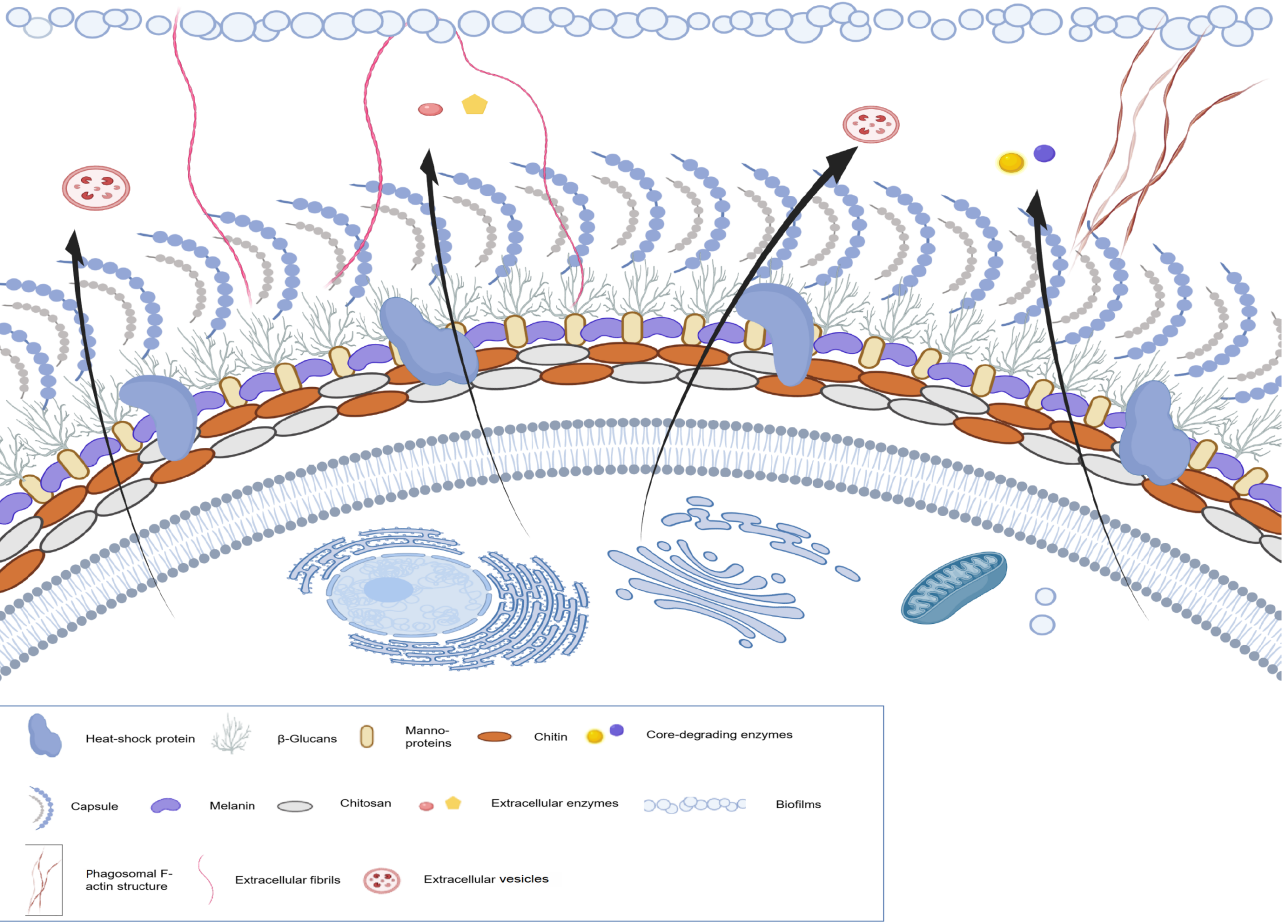
Figure1 Traditional virulence factors and some potential novel virulence factors These factors include capsule, melanin, chitin, chitosan, the ability to grow at physiological temperature, extracellular enzymes, extracellular vesicles, heat-shock protein, core-degrading enzymes, biofilms, the phagosomal F-actin structure, and extracellular fibrils.

Previous studies have shown that the capsule, melanin, chitin，chitosan growth ability at physiological temperature, degrading enzyme [ 3, 4, 31] and some potential novel virulence items are closely related to the pathogenicity of *Cryptococcus* ( Figure 1).

### Capsule

The capsule (CP) consists of 90%–95% glucuronoxylomannan (GXM), 5% galactosexyylomannan and less than 1% mannoprotein, which is required for the survival of *Cryptococcus* in its host [32]. In addition to the three conventional ingredients mentioned above, minor and lesser capsular components have also been identified in *C*. *Neoformans*, including heat-shock proteins [33], glucans [34] and chitooligomers [35]. Genes associated with the capsular formation and their functions have been reported in *C*. *Neoformans*, including *CAP59* (a transmembrane protein that mediates GXM transport), *CAP64* (and *CAP60*/ *CAP10*, proteins encoded in the nuclear envelope and cytoplasm), *CAS1*/ *CAS3* (mediating GXM acetoylation), *UXS1*/ *UGD1*/ *CAS31*/ *CAS32*/ *CAS33*/ *CAS34*/ *CAS35* (mediating xylosylation of GXM) [31], but whether they also play an important role in *C*. *gattii* is less studied. Then how do the capsule and its constituents cause disease? At the physical level, the polysaccharide capsule provides a physical barrier that can interfere with the normal phagocytosis and clearance of macrophages in the immune system [1]. And *Cryptococcus* can initiate the expansion of its polysaccharide capsule and alter the composition of the capsules (structure, density, and size) dynamically to optimize survival opportunities, depending on environmental conditions [ 36, 37]. At the immune interference level, *Cryptococcus* releases polysaccharides from their capsule into the periphagosomal vesicles in macrophages and they are accumulated in the cytoplasm of host cells, leading to macrophage dysfunction and lysis [ 3, 4]. Mannoproteins (MPs) affect the thickness and phagocytosis of *C*. *gattii* cell-associated cryptococcal polysaccharides, and the absence of MPs disturbs the size distribution of podosomal polysaccharides, resulting in a scattered distribution [32]. A previous study showed that GXM inhibited the activation of *Cryptococcus* mannoprotein-specific hybridoma T cells and the proliferation, antigen uptake, and processing of OVA-specific OT-II T cells. Moreover, GXM directly inhibits T cell proliferation induced by CD3 antibodies, concancanin A, or phorboll-12-myristic acid-13-acetate/iamycin [38]. It has been reported that encapsulated *C*. *gattii* blocks DC maturation-dependent extracellular receptor signaling by TNF-α and P38 MAPK, which results in a defective T cell response [39]. Another study demonstrated that CPs support immune evasion by coating CD11b antigen and blocking CD11b interaction with *C*. *gattii* cells, so that DCs cannot recognize *C*. *gattii* capsular cells [40]. In summary, the capsule can be pathogenic by forming a physical barrier and interfering with the host immune response.

### Cell wall

The cell wall is composed of a matrix of glucose (Glc), *N*-acetylglucosamine (GlcNAc) and glucosamine (GlcN) polymers (glucan, chitin and chitosan, respectively) with covalently and noncovalently associated glycoproteins [41]. At present, the study of cell wall in *C*. *gattii* is mainly about melanin, chitin and chitosan.

#### Melanin

Melanin is a negatively charged hydrophobic high molecular weight pigment that is formed by oxidative polymerization of phenolic compounds [42]. In the presence of certain catechol compounds such as 3,4-dihydroxyphenylalanine, laccase catalyzes melanin synthesis in *Cryptococcus* spp [43]. Laccase enzyme is required for the biosynthesis of melanin [44]; therefore, its activity has been reported to be associated with enhanced survival of the fungus in macrophages [45]. Two laccase genes, *LAC1* and *LAC2*, were identified as central enzyme genes for melanin biosynthesis [31]. Another study on the highly virulent *LAC1*-deficient *C*. *gattii* strain R265 showed that laccase expressed by *C*. *gattii* may promote pulmonary fungal growth by inhibiting the induction of Cryptococcal-specific IL-17 cytokine response and the recruitment and function of neutrophils, indicating its role in regulating fungal uptake, intracellular survival, and macrophage killing. This is similar to what was observed in *C*. *neoformans* [46]. Related studies have found a similar relationship in immunocompetent goats [47]. Other genes, including *VPH1*, *CLC1*, *CCC2*, *ATX1* and *MBF1*, have also been found to be necessary for melanin production [31].

#### Chitin and chitosan

An important component of the fungal cell wall that contributes to its strength and integrity is chitin, which is a linear polymer of β-(1,4)-linked *N*-acetylglucosamine (GlcNAc), formed from the cytoplasmic pool of UDP-GlcNAc [48]. Chitosan is one of the carbohydrate polymers in the cell wall that significantly affects the outcome of host-pathogen interaction [49]. In *C*. *neoformans*, the conversion of chitin to chitosan is catalyzed by chitin deacetylases 1–3 ( *Cda1*, *Cda2*, and *Cda3*) [50]. A previous study found that defects in the *Cda3* gene coding for *C*. *gattii* exhibited cell wall damage and reduced virulence, whereas *Cda1* defects had no effect on the virulence of different mouse strains of *C*. *gattii* R265 [51]. However, deletion of *Cda3* gene alone or in combination in R265 did not affect its ability to produce capsule or melanin, suggesting that *Cda3* is a specific gene of *C*. *gattii* cell wall, and its encoded enzyme protein Cda3 is important for cell wall chitosan production and can influence virulence [51].

### The ability to grow at physiological temperature

The ability to grow at physiological temperature is essential for the virulence of *C*. *gattii* and *C*. *neoformans*. Although some *Cryptococcus* species also have capsules and produce melanin, they rarely grow at 37°C and therefore do not cause infection in mammals. Previous studies have identified many genes that are essential for high-temperature growth in *C*. *neoformans* and *C*. *gattii*, including *CNA1*, *CNB1*, *CPA1*, *CCN1*, *TPS1*, *TPS2*, *MGA2*, *RAS1*, *SOD2*, *TSA1*, *ILV2*, *SPE3*/ *LYS9*, *MPK1*, and *STE20*, etc [52].

### Extracellular enzymes

The secretion of active extracellular enzymes, such as proteases, phospholipases, and ureases, contributes to virulence by destroying and degrading host molecules as a means of obtaining nutrients, countering immune responses, and spreading throughout the body [53]. Proteases degrade host proteins including collagen, elastin, fibrin, immunoglobulin and complementary factors, causing destruction of host tissues and providing nutrients for *Cryptococcus* [54]. Phospholipases enhance adhesion to lung epithelial cells and hydrolyze phospholipid ester linkages to penetrate host tissues [ 55, 56]. Wright *et al*. [57] demonstrated the presence of two secreted phospholipase proteins, PLB1 and a new LPL1 in *C*. *gattii*, whose molecular weight (670 kDa) was found to be very large, with an isoelectric point of approximately 6.2 on IEF/PAGE, as measured by size-exclusion chromatography. The activity of LPL1 at 70°C may be the reason for the predominant distribution of *C*. *gattii* in tropical and subtropical regions. Urease (EC 3.5.1.5) is encoded by the gene *URE1* and Ni ^2+^-dependent metalloenzymes produced by plants, fungi and bacteria that hydrolyze urea to produce ammonia and CO _2_ [58]. Knockout mutants of the urease-encoding gene *URE1* and the coproteins Ure4 and Ure6 reduce intracellular proliferation in macrophages, while in nasal infected mice, ure1D (urease protein deficiency) and ure4D (apo urease with inactivated enzyme activity) mutants result in reduced blood load and delayed time to death [59]. Urease positive strains result in reduced survival and increased dissemination to the brain in mice compared with urease-negative strains [60]. These examples all suggest the role of urease in the virulence of *C*. *gattii*.

### Novel virulence phenotype

Although the virulence above has been confirmed before, there is still much room for exploration in *C*. *gattii*. In recent years, researchers have identified some potential virulence factors that may extend the understanding of *C*. *gattii* virulence, but further studies are needed to confirm the target genes and regulatory mechanisms.

#### Extracellular vesicles

Extracellular vesicles (EVs) are membranous structures produced by prokaryotes and eukaryotes, including 14 fungal genera. Among fungi, they were first characterized in culture fluids of *C*. *neoformans* [61]. Detailed analysis of the composition of EVs from a range of pathogenic fungi revealed a wide spectrum of RNAs, short non-coding RNAs, ribosomal proteins and proteins associated with virulence, antioxidant defence and pathogenesis that may contribute to yeast survival and proliferation [ 62, 63]. A decade later, *C*. *gattii* was also shown to produce EVs. EVs released by *Cryptococcus* occur in distant locations, and these vesicles spread throughout the body and are rapidly internalized by macrophages, leading to an increase in the proliferation rate of *Cryptococcus* during phagocytosis. The experimental data also confirmed that the presence of the capsule may enhance the virulence of EVs, and EVs exert their virulence mainly through RNA and protein protected by lipid bilayer [64]. Taken together, these results suggest that *C*. *gattii* can use EVs to communicate and coordinate information between cells to improve survival in the host.

#### Heat-shock proteins

Heat-shock proteins (Hsps) are chaperones that play an important role by helping other proteins to achieve their 3D conformation and are recognized as one of the components of secretory vesicles in *C*. *neoformans* [65], and Hsp70 was recently proved to modulate the interaction between *C*. *neoformans* and human-type alveolar epithelial cells, and to decrease fungal killing by mouse macrophages [33]. By inhibiting Hsp90, biofilm formation, membrane permeability, protein release and cell survival at 37°C in *C*. *gattii* are reduced, but melanin production is not [66].

#### Core-degrading enzymes

Core-degrading enzymes are essential components of the basic secretion body of *Cryptococcus*, including polysaccharide-active enzymes, glycoside hydrolases, carbohydrate esterases, and polysaccharide lyases that degrade major components of plant cell walls such as cellulose and pectin [67]. Some proteins secreted by *C*. *gattii* VGIIb strain have homologous that can initiate the host immune responses and prevent disease, including the glycolytic proteins glyceraldehyde-3-phosphate dehydrogenase (E6R7D5), enolase (Q55UX4), and 6-phosphogluconate dehydrogenase (E6RDR8) and the stress response protein Cu-Zn superoxide dismutase (Q9C0S4). Conversely, hypervirulent strains lacking these secreted immune-stimulating proteins may evade host immune detection, resulting in pulmonary infections, and possibly facilitating transmission to the central nervous system [68].

#### Biofilms

Biofilms are microbial communities of extracellular matrix on solid surfaces, which increase the concentration of nutrients in the biofilm-liquid interface and provide protection from environmental damage [69]. *C*. *gattii* forms a highly organized and complex biofilm on abiotic surfaces and significantly reshapes the transcriptome profile during biofilm formation, while up-regulated genes related to information processing, stress response, utilization and transcript in the cell-to-cell adhesion are detected, which are closely related to metabolism, growth and pathogenicity [70]. Genetically, increased expressions of *LAC1* and *URE1* genes are associated with *C*. *gattii* biofilm and virulence [69].

#### The phagosomal F-actin structure

The phagosomal F-actin structure is retained by the hypervirulent *C*. *gattii* strain and blocks the phagocytosis of dendritic cells. Super-resolution structural lighting microscopy (SR-SIM) revealed that the persistent phagosome F-actin forms cage-like structures that spatially and functionally obstruct lysosome fusion. Blocking lysosome fusion is sufficient to inhibit phagosomal acidification and subsequent DC killing of intracellular fungi [71]. *C*. *gattii* strains that retain phagosomal F-actin can also cause DC immune paralysis. Destruction of retained F-actin cage with cytolaxin D not only restores DC phagosomal maturation, but also promotes DC costimulatory maturation and intense T cell activation and proliferation [71].

#### Extracellular fibrils

Extracellular fibrils of *C*. *gattii* are 40–100 nm in diameter and 500– 3000 nm in length, whereas capsule-deficient *C*. *gattii* mutants are completely devoid of extracellular fibrils. In mouse lung models and in systemic cryptococcal disease, extracellular fibrils in *C*. *gattii* are more virulent than those without fibrils. *In vitro*, *C*. *gattii* cells with extracellular fibrils are also significantly more resistant to human polymorphonuclear neutrophils (PMNs). These observations suggest that the formation of extracellular fibril formation may be a structural adaptation of *C*. *gattii* cell-cell, cell-substrate, and/or cell-phagocytic communication that initially enhances virulence in mammalian hosts by inhibiting host PMN-mediated killing [72].

## Traditional Stress Signaling Pathways and Some New Regulation Mechanisms of *C*. *gattii*

Advances in the genomics revolution have revealed a complex array of genes and their transcripts that contribute to the study of the virulence complex of *C*. *gattii* [4]. Bahn and Jung [73] provided a comprehensive summary of stress signaling pathways and the pathogenicity of *Cryptococcus* that act through multiple stress signaling pathways through a panoply of signaling components, including receptors/sensors, small GTPases, secondary messengers, kinases, transcription factors, and other miscellaneous adaptors or regulators [73]. Through deletion mutants, traditional genes, transcription factors, and signaling molecules are associated with virulence of *C*. *gattii* and *C*. *neoformans* [ 74– 86] ( Table 1).

**Table:** **Table1** Traditional genes, transcription factors, and signaling molecules that have been linked to virulence in *C*. *gattii*

Gene(s)	Function		Phenotype		Required for virulence, model
*SOD1*	Cytoplasmic antioxidant		Required for production of virulence factors urease, PLB, and laccase in *C*. *gattii* but not *C*. *neoformans*		In BALB/c mouse intravenous inoculation model and in A/JCr mouse inhalational model of *C*. *neoformans*
*SOD2*	Mitochondrial antioxidant		Required for growth at 37°C in 20% but not in 1.3% oxygen		For *C*. *gattii* BALB/c mouse inhalation and i.v. inoculation model; *C*. *neoformans* was not tested
*TPS1*, *TPS2*	Trehalose biosynthesis; trehalose functions as an antioxidant and stress protectant		Required for thermotolerance, capsule and melanin production, mating, and cell wall integrity in *C*. *gattii* and thermotolerance in *C*. *neoformans*		For *C*. *gattii*, *Caenorhabditis elegans* (worm) and A/JCr mouse inhalational models; TSP1 but not TSP2 is required for virulence in *C*. *neoformans*
*PKA1*, cAMP	Signal transduction pathway regulator		Required for capsule production in *C*. *gattii*,mating and capsule and melanin production in *C*. *neoformans*		*C. gattii* was not tested; yes, for virulence of *C*. *neoformans* in BALB/c mouse inhalational model and immunosuppressed rabbit CSF inoculation model
*PKA2*, cAMP	Signal transduction pathway regulator		Required for mating and capsule and melanin production in *C*. *gattii* but not *C*. *neoformans*		Not tested
*PLC1*	Signal transduction pathway regulator		Regulates growth at 37°C and melanin and PLB production in *C*. *neoformans* through the PKC/MAPK pathway (see below)		In *C*. *neoformans* BALB/c mouse inhalational model
*MPK1*	Signal transduction pathway regulator		Regulates melanin, capsule production, and cell wall integrity in *C*. *gattii* and thermotolerance and cell wall integrity at 37°C in C. *neoformans*		*In *C**. *gattii* (BALB/c inhalational model) and in *C. neoformans* i.v. DBA/2 complement-deficient mouse model
*STE12* **α**	Transcription factor		Regulates melanin, mating, and ecological fitness in *C. gattii* and regulates mating and capsule size in *C. neoformans*		In *C*. *gattii* but not *C*. *neoformans*
*GAT1*	GATA transcription factor		Regulates nitrogen utilization		In *C*. *gattii* but not in *C*. *neoformans* BALB/c intrapharyngeal instillation model
*CNA1*	Subunit of the heterodimer calcineurin, a Ca ^2+^ calmodulin-activated serine-threonine-specific protein phosphatase		In *C*. *gattii*, regulates thermotolerance (37°C) (strains differ) and is required for plasma membrane integrity, tolerance to fluconazole, and optimal growth in the presence of Ca ^2+^and Li ^+^, with no role in melanin and a minor role in capsule production; in *C*. *neoformans*, lesser effect of Ca ^2+^ and not required for fluconazole tolerance		In *C*. *gattii* (molecular type-dependent), *G. mellonella* (wax moth) larva and A/JCr mouse inhalational models and also in *C*. *neoformans* rabbit intracisternal inoculation and BALB/c mouse i.v. inoculation models

In addition to the currently known traditional pathways, many new studies have been carried out to identify additional ways in which genes affect virulence ( Table 2).

**Table:** **Table2** Some potential regulation mechanisms

Gene(s)		Function		Phenotype		Required for virulence, model
*DAOs*		D-amino acid		Growth abilities/melanin production		In BALB/c mouse intravenous inoculation model; *C. neoformans* was also tested
*CAN1*		β-carbonic anhydrase (β-CA)		CO _2_ concentration in growth environment		For *C. gattii* BALB/c mouse inhalation model; *C. neoformans* was not tested
*CAPs*		Chitooligomer, chitinase		Capsule production		For *C. gattii* BALB/c mouse inhalation model; *C. neoformans* was not tested
*ZAP1, ZAP2*		Transcription factors		Zinc utilization		For *C. gattii* BALB/c mouse inhalation model; *C. neoformans* was not tested
*ZAP3*		Cytoplasmic antioxidant		Growth abilities/capsule production		Galleria mellonella larvae intravenous inoculation model; *C. neoformans* was not tested
*UbP5*		Ubiquitination and deubiquitination/copper ion metabolism or polysaccharide attachment to the cell wall		Tolerance to multiple stressors/melanin and capsule production		For *C. gattii* BALB/c mouse inhalation model; *C. neoformans* was not tested
*CDA3*		Chitosan		Cell wall integrity		For *C. gattii* CBA/J mouse inhalation model; *C. neoformans* was not tested
*CNVs*		Metabolism, energy and biological stimulus-response in transmembrane transport, methylation, transport and related proteins		?		No model
*MFE2*		Peroxisome multifunctional enzymes/Regulates fatty acid β-oxidation metabolism		?		For *C. gattii* CBA/J mouse inhalation model; *C. neoformans* was not tested
*FAS1, FAS2*		Transcription factors/regulation of fatty acid β-oxidation metabolism		?		For *C. gattii* CBA/J mouse inhalation model; *C. neoformans* was not tested
Gene of some specific transcription factors		Transport protein and amino acid metabolic pathways		?		For *C. gattii* CBA/J mouse inhalation model; *C. neoformans* was not tested

### *DAO* genes in growth abilities/melanin production

Most *C*. *gattii* and *C*. *neoformans* strains take D-proline or D-alanine as the only nitrogen source, and absorb D-tryptophan to produce pigment. Triple deletion of D-amino acid oxidase genes ( *DAO1*, *DAO2*, and *DAO3* with *DAO2* as the major gene) causes the deficiency of D-amino acid, alters the nitrogen source, leads to impaired growth capacity and melanin production, and affects the virulence [87].

### *CAN1* gene in CO _2_ concentration in the growth environment

Genetic analysis of β-carbonic anhydrase (β-CA) encoded by *C*. *gattii* *CAN1* and *CAN2* showed that the *CAN2* mutant, in contrast, exhibits a severe growth defect in ambient air, but not in a high-CO _2_ environment, so *CAN2*, but not *CAN1*, is the main β-CA gene [88].

### *CAP59* and *CAP60* in capsule production

The *C*. *neoformans* strain ATCC 24066 phenotype is accompanied by decreased expression of *CAP59*, which was predicted to encode the protein required for GXM export, and brain infection is actually associated with increased distribution of fungal cell surface chitooligomer *in vivo* and elevated chitinase activity in the lungs of infected mice [89]. While in *C*. *gattii*, the mechanism is unclear. The decreased expression of *CAP59* and enhanced expression of *CAP60* was enhanced in VGIII isolates, which had smaller capsule diameter and lower virulence, compared with VGIV isolates *in vitro*, suggesting that *CAP59* and *CAP60* may play an important role in *C*. *gattii* virulence [6].

### *ZAP1* and *ZAP2* in regulating zinc utilization and *ZAP3* in regulating growth abilities/capsule production

Zinc homeostasis is essential for fungal growth, both as a catalytic constituent and as a core component in the structure. Zinc deficiency induces decreased growth in all strains compared to those grown in the zinc-rich medium [90], but different studies differed in the analysis of zinc-related genes. *ZAP1*, a gene of zinc finger protein, regulates the expressions of several genes involved in zinc metabolism. The data indicated that the relative fluorescence level of zinc content in the zap1Δ mutant strain was about 5-fold lower than that in wild-type (WT) strain, and the transcript levels of two genes ( *Zip1*-CNbG_6066 and *Zip2*-CNbG_2209) were significantly lower than those in WT cells as detected by qRT-PCR [91]. Another part of the data showed a significant increase in reactive oxygen species (ROS) accumulation in zap1Δ mutated cells after culture on TPEN-rich YNB medium, compared to that in WT and supplementary strains. The results of this study suggest that *ZIP1* is necessary for key events in the mechanisms associated with cryptococcal disease [91]. Reduced virulence of *C*. *gattii* *zip1Δzip2Δ* double deletion mutants was observed in intranasal infection in mice, mainly due to decreased expressions of gene-related transcription factors, leading to decreased zinc uptake, decreased zinc concentration and increased intracellular ROS. So these studies confirmed the importance of proper zinc uptake to cryptococcal virulence [92]. The *ZIP3* gene of *C*. *gattii* can encode a manganese transporter localized to the Golgi apparatus membrane. Zip3-deficient cells are tolerant to toxic concentrations of manganese and have an imbalanced expressions of intracellular metal transporters, such as the vacuolar Pmc1 and Vcx1, as well as the Golgi Pmr1. Deletion mutants of the *ZIP3* gene are more sensitive to ROS. More importantly, the *ZIP3* null mutant strain displayed decreased melanization and secretion of the major capsular component GXM, as well as an altered extracellular vesicle dimensions profile [93].

### *Ubp5* in tolerance to multiple stressors, melanin and capsule production

*C*. *gattii* strain R265 is highly tolerant to a variety of pressure sources *in vitro*, but *ubp5Δ* mutant ( *Ubp5* deletion) exhibits high-temperature sensitivity, partial growth defects at 37°C, and complete inhibition at 39°C. Partial growth defects were also observed after exposure to osmotic shock or cell membrane/wall damaging agents. Many misfolded or damaged proteins are accumulated in *Cryptococcus* cells, a phenomenon that depends partly on the ubiquitin-mediated degradation pathway to maintain cell homeostasis. Ubiquitination and deubiquitination in the ubiquitin-proteasome pathway may be important modification mechanisms for some signaling pathways related to fungal stress responses. At the same time, the *ubp5Δ* mutant strain significantly increases the capsule and melanin production, which may be related to its role in regulating copper metabolism or polysaccharide attachment to the cell wall [94].

### *Cda3* in cell wall integrity

The previous results showed that the cytoderm of *C*. *gattii* R265 contained two to three times more chitosan than that of *C*. *neoformans*, where the role of chitin deacetylases ( *Cdas*) was gradually gaining attention. The haploid, diploid and triploid *Cda*-deficient strains were constructed in the context of R265, and the results showed that *Cda3* was very important for the virulence of *C*. *gattii*, unlike *Cda1* in *C*. *neoformans*. Deletion of *Cda3* alone or in conjunction with another *Cda* ( *Cda1Δ3Δ* or *Cda2Δ3Δ*) or both ( *Cda1Δ2Δ3Δ*) rendered the fungus avirulent and cleared from the infected host. These studies begin to illuminate the regulation of chitosan biosynthesis of *C*. *gattii* and its subsequent effect on fungal virulence [51].

### Copy number variations

Several potential virulence-associated genes were found in high V _ST_ regions. Researchers did identify copy number variations (CNVs) in several genes related to transport activity by GO term enrichment analysis on all genes in the high V _ST_ *CNV* regions. Statistically different GO terms were enriched in some metabolic processes. For example, the top three GO terms enriched in the biological processes category were transmembrane transport, methylation, and transport. In the category of biological processes, the enrichment of protein domains associated with primary metabolisms, such as carbohydrate metabolic process, telomere maintenance, and mitochondrial proton-transporting ATP synthase, catalytic core was also observed. These results suggest that CNVs can affect metabolism, energy and biological stimulus-response in transmembrane transport, methylation, transport and related proteins, and affect the virulence and pathogenicity of *C*. *gattii*. Further functional investigations of candidate genes are needed to better understand the impact of copy number variation on *C*. *gattii* virulence [95].

### *MFE2*, *FAS1* and *FAS2* in regulating fatty acid β-oxidation metabolism

The development of microarray data analysis, transcriptomics and metabolomics has promoted the big data research of gene phenotypes, virulence phenotypes, related pathways and influencing factors. Based on big data, researchers can stand on the shoulders of giants to further refine relevant research. Microarray analysis showed that the transcription levels of genes involved in fatty acid β-oxidation differ among *C*. *gattii* strains. The multifunctional enzyme encoded by the peroxisomal multifunctional enzyme 2 ( *MFE2*) gene catalyzes the second and third reactions in peroxisome in fatty acid β-oxidation, and is an important virulence factor for growth. Similarly, fatty acid synthase genes ( *FAS1* and *FAS2*) are differentially regulated by transcription in *C*. *gattii*, but the specific pathway is unclear and needs further analysis [25].

### Genes of some specific transcription factors

Transcriptional analysis performed on data from bronchoalveolar lavage fluid in mice infected with *C*. *gattii* hypervirulent strain R265 showed that about 85 transcription factors present higher abundance expression, and enrichment of downstream transport protein and amino acid metabolic pathways, especially the transcription of biodegradable material concentration is more obvious than synthesis-related transcription, such as the *GATA* gene involved in the regulation of nitrogen metabolism, the ammonium permease gene mediating the absorption of ammonium, and the cytoplasmic amino acid permease genes AA4 permease *Amt1* and *Amt2* encoding the APC (amino acid-polyamine-choline transporter) superfamily. It suggested the existence of some novel highly expressed genes and amino acid metabolism pathway that allow *C*. *gattii* to proliferate and survive *in vivo* [96].

## The Opposite Point of View of the Relationship between Gene and Virulence

An increasing number of studies have shown that some specific transcriptional and metabolism-related substances can influence *C*. *gattii* virulence. For example, there is still much space to explore in terms of biosynthesis, remodeling, oxidation and so on, while other researchers take a different view. Firacative *et al*. [97] tested the WGS and rearrangement comparative analysis of specific genome-wide or mitochondrial genes in hypervirulent and hypovirulent VGIII strain, and their results did not show any specific change, suggesting that there are no specific genome-wide or mitochondrial genome differences between hypervirulent and hypovirulent VGIII strains. Another study also confirmed that in highly pathogenic *C*. *gattii* strains of all major molecular types, virulence was found to be independent of the major molecular types, but related to different characteristics of individual strains, indicating that virulence depends on different structural elements and the regulatory expressions of multiple factors, as well as the complex multi-genetic traits of pathogenic microorganisms, which may contain some unrecognized unique characteristics [20]. These findings highlight the urgent need to combine genomic, transcriptomic, and metabolomic approaches to characterize additional characteristics of *C*. *gattii* which may be a more reliable predictor of pathogenicity than the accepted virulence indicators.

## What Are the Characteristics of Hypervirulent *C* **.** *gattii* Strains?

As shown above, the hypervirulent strains of *C*. *gattii* are often the result of a mixture of multiple factors. For example, the presence of *LAC1*, *SOD1* and *MPK1* genes suggests the possibility of hypervirulent strains. Meanwhile, microevolution of genes, such as rearrangement and mutation, can lead to the emergence of hypervirulent strains. It has been suggested that the environment of *C*. *gatti* and the changes of hormones and metabolic substances in patients can also enhance the virulence of *C*. *gattii* [ 98– 101], but research data are scarce. Additionally, virulence factors are regulated by many factors in the process of gene expression, so the transcription and metabolism of hypervirulent strains also suggest certain specificity. Therefore, it is difficult to define specific markers for highly virulent strains, and multiple markers are needed to determine them together.

## Conclusion

Previous studies are necessary to understand the complex gene-virulence patterns and stress signaling networks, which can help prevent and control *C*. *gattii* outbreaks and develop new antifungal therapies. There is still much room for further exploration in *C*. *gattii* gene, virulence and connection pathway by exploring the traditional stress signaling pathway as well as some new ones. However, despite mounting evidence of extensive crosstalk between multiple stress signal cascaded pathways, future research needs to focus on how the pathways are coordinated in response to incoming stress, host-pathogen interactions, and the development of useful models that accurately reflect human disease phenotypes. We also need to better understand the pathways from genetic variation to phenotypic variation and ultimately disease characteristics in *Cryptococcus* species.
